# Update and Reassessment of Data on the Role of Osteocalcin in Bone Properties and Glucose Homeostasis in OC-/- Mice

**DOI:** 10.3390/ijms27010170

**Published:** 2025-12-23

**Authors:** Steven Tommassini, Terry Lynne Dowd

**Affiliations:** 1 Departments of Orthopaedics and Rehabilitation, Yale University School of Medicine, New Haven, CT 06510, USA; 2 Department of Chemistry, Brooklyn College of the City University of New York, Brooklyn, NY 11210, USA; 3 PhD Program in Biochemistry, The Graduate Center of the City University of New York, New York, NY 10012, USA

**Keywords:** osteocalcin knock-out mouse, osteocalcin, diabetes

## Abstract

Osteocalcin (OC) is a small protein (49 aa) produced by the osteoblast and the most abundant noncollagenous protein in bone. Studies using the osteocalcin-depleted knock-out mouse (OC-/-) reported that osteocalcin affects bone mineral properties and bone strength. Other reports indicated osteocalcin was a hormone regulating glucose metabolism by increasing insulin secretion and sensitivity. These results were controversial. Several years ago, a couple of groups generated osteocalcin knock-out mice using different methods and failed to observe an effect of osteocalcin on bone mineral properties, bone strength or glucose metabolism. One review claimed that the previously reported results from OC-/- mouse studies were inconsistent. Within the last 2 years, additional OC-/- mouse studies have been reported, and they confirm the role of osteocalcin in glucose metabolism, bone mineral properties and strength. Some of the new data reported clears up any controversy. Published studies on the role of osteocalcin in bone and glucose metabolism using OC-/- mice will be reviewed, and results will be compared. It is shown that most of the data on the effect osteocalcin on bone properties and strength is consistent and corroborated when comparing valid data from similar techniques and similar regions of the bone. Data corroborating the role of osteocalcin in glucose metabolism will be presented, and reasons for conflicting results will be discussed. Diabetics and the elderly have reduced osteocalcin levels and are prone to bone fractures, while diabetics are glucose-intolerant. Osteocalcin may be of therapeutic use to these populations.

## 1. Introduction

Osteocalcin (OC), a small protein (46 amino acids long in mice and 49 amino acids long in humans) is the most abundant noncollagenous protein in bone [1,2]. It has been reported to be a hormone regulating glucose homeostasis and also shown to play a role in bone strength and fracture resistance. Studies on osteocalcin-depleted mice (OC-/-) are relevant for diabetes since diabetics also have reduced osteocalcin, elevated blood glucose and are prone to fractures like the OC-/- mice.

Several different groups have reported results on the role of osteocalcin in bone mineral properties and on the role of osteocalcin on glucose homeostasis using OC-/- mice [3,4,5,6,7,8,9,10]. These will be discussed in this review. Some of the results were conflicting, giving the appearance of irreproducibility. However, upon examination, many of the bone properties reported comparisons of wild-type (WT) vs. OC-/- mice without considering background or age differences. The results were labeled as conflicting but instead were due to misinterpretation of the parameters being measured and incorrect comparison from different techniques or from different regions of the bone. Also, within the past year, a couple of recent publications significantly shed light on the conflict concerning the role of osteocalcin in bone and as a glucose-regulating hormone, which cleared up some of the controversy. In this review, we will discuss the results concerning the role of osteocalcin in bone properties, bone strength and glucose regulation by focusing on new and appropriate OC-/- vs. WT mouse studies. Inconsistent results will be explained, and it will be shown that much of the data between OC-/- mice is corroborated.

**A.**  **Structural studies**

**Figure 1:**
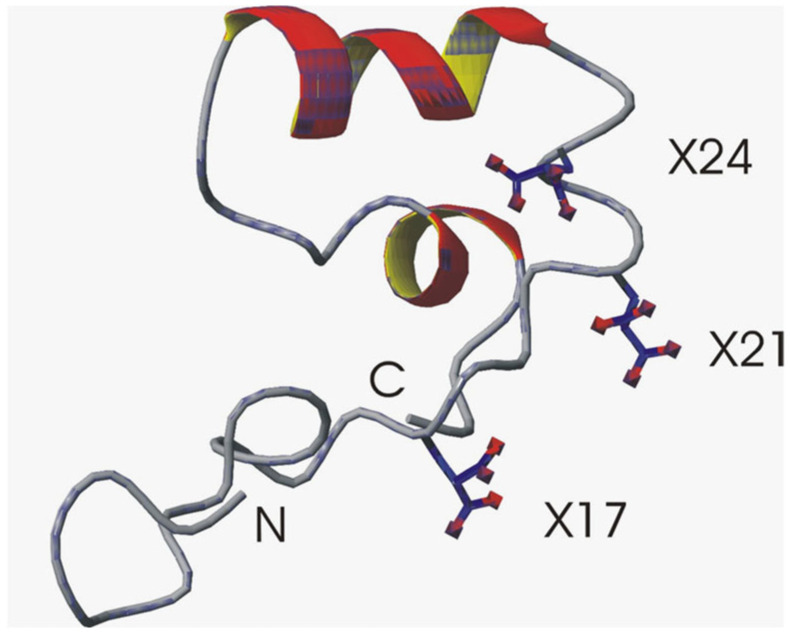
1H NMR: Ca^2+^ 3 Gla-OC (residues 1-49) [13]. N, C–N and C termini respectively. X—Gla residues.

**Figure 2:**
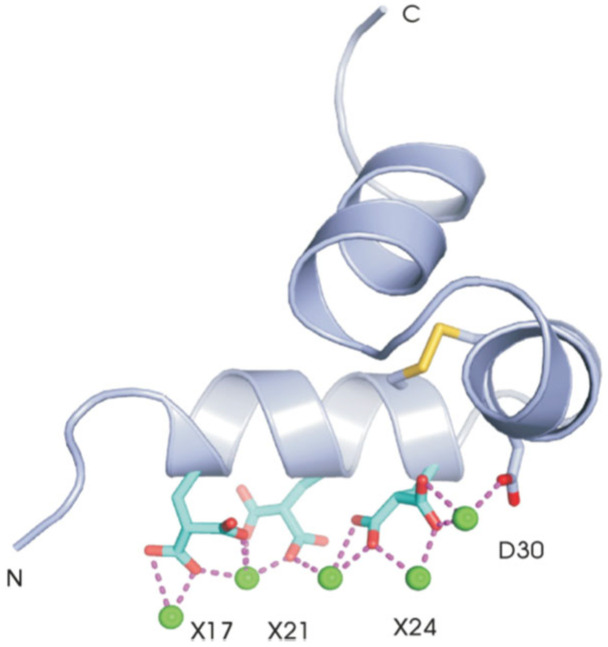
X-ray Crystal structure: 3 Gla Ca^2+^ -OC (residues 15-49) [14,20]. X—Gla residues, D—aspartic acid, green balls are Ca^2+^ ions, red dotted lines—coordinating O atoms.

Osteocalcin is synthesized by osteoblasts as the 3 Glu-osteocalcin form and then post-translationally modified to 3 Gla-osteocalcin (γ-carboxyglutamic acid) in a vitamin-K-dependent carboxylation of the 3 glutamic acid residues [11]. Early structural studies using circular dichroism found metal-free osteocalcin existed in a random coil where millimolar concentrations of Ca^2+^ produced a conformational change to a partial α-helical conformation [12]. This was confirmed by a 2D ^1^H NMR high-resolution structure of Ca^2+^- osteocalcin, which displayed an unstructured N-terminus (residues 1-16) followed by a more structured helical region (residues (15-49), which included the 3 Gla residues (Figure 1) [13]. The structured portion of Ca^2+^-osteocalcin was later solved by X-ray crystallography (residues 17-49), and it was reported that osteocalcin has three helical regions and a hydrophobic core (Figure 2). There were three tight and two weaker Ca^2+^ binding sites, which confirmed the Gla residues can bind Ca^2+^ and were separated by distances which were complimentary to Ca^2+^ ions in the bone crystal [14] (Figure 2). Therefore, in vivo osteocalcin is mainly bound to bone mineral with lower concentrations in the serum [15,16,17,18,19].

**Figure 3:**
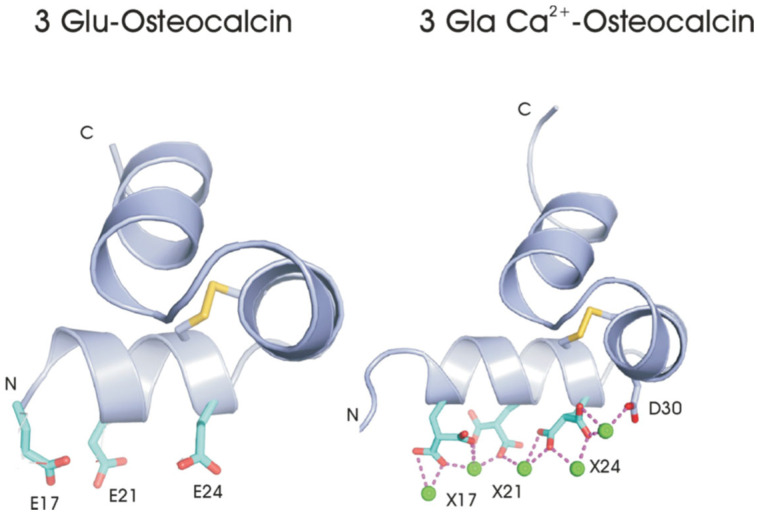
[20] X-ray Crystal Structures: 3 Glu-OC and Ca^2+^ 3 Gla-OC. E—Glutamic acid, X—Gla residues, green balls representing Ca^2+^ ions and red lines showing coordinating O atoms.

Later studies (discussed in a subsequent section) reported osteocalcin functions as a hormone regulating glucose metabolism [6,7]. The uncarboxylated (3 Glu-OC) or undercarboxylated (1 Glu-OC) forms of osteocalcin are the potential regulators of glucose metabolism [6,7]. The 3 Glu-OC structure was solved with X-ray crystallography and found that the apo- (Ca^2+^-free) form of the 3 Glu-OC form had a very similar structure to 3 Gla Ca^2+^-OC [20] (Figure 3). It was suggested that the 3 Glu-OC may be involved in cell receptor interactions where helical osteocalcin is required and the Ca^2+^ concentration is low, while 3 Gla Ca^2+^-OC affects bone and mineral crystal properties through its ability to bind divalent metal cations in both mineral and solution [20].

## 2. OC-/- Mice

There are three genes, *Bglap*, *Bglap2* and *Bglap3*, which form the mouse osteocalcin gene cluster, with one gene, *Bglap*, being identified in rats and humans [21,22,23]. Two of the genes, *Bglap* and *Bglap2*, encode osteocalcin and are expressed only in osteoblasts in bone. *Bglap3* is expressed in the kidneys, lungs and male gonadal tissues. A number of OC-/- mice were produced using different methods and different genetic backgrounds. The genetic background of a mutant mouse refers to all of its genetic make-up except the mutated gene of interest. Within this genetic make-up are modifier genes which may interact with and modify the phenotype or expression of a mutation [24]. The phenotypic variation can be major or minor and can also be shown in different phenotype onset times. Effects of genetic background on phenotypic variation can also be seen in humans with the same mutation in the identical gene but differ in the number of other modifier genes [24].

The Karsenty group generated the first OC-/- mouse by deleting both *Bglap* and *Bglap2* concurrently using embryonic stem cell technology [5]. With both these genes deleted, osteocalcin is not expressed in the mice. These mice were on a 129/BL6 mixed genetic background. Two other independent labs used female or male 129/BL6 OC-/- backcrossed to a C57BL/6J background (10 generations) [8,9,10,25,26]. Komori et al. also created an OC-/- mouse by deletion of *Bglap* and *Bglap2* using gene targeting in embryonic stem cells and backcrossing to a C57BL/6N background (8–10 times) [3]. An OC-/- mouse was also created with a *Bglap* and *Bglap2* double knock-out allele using CRISPR/Cas9-mediated gene editing [4] and had a mixed genetic background C57BL/6J;C3H. However, there have been a number of problems reported with the methodology involved in producing this mouse. Among the problems reported were the presence of mutations in the sequence of WT controls and only two generations of backcrossing, which would prevent the elimination of off-target mutations [27]. A very recent study found blatant obesity restricted to the wild-type mouse due to over 11,000 insertions or deletions over 50 bps long and over a million smaller length variants in the wild type compared to other wild-type mice generated by different methods and on different backgrounds [28]. Due to the obesity of the WT mice, results from both bone mineral studies and glucose metabolic studies obtained from the CRISPR/Cas9 OC-/- mice [4] cannot be considered or contrasted with other OC-/- mouse studies.

## 3. Effect of Osteocalcin on Bone Quality

Many in vitro and in vivo studies have reported functional properties of osteocalcin. In vitro studies have shown osteocalcin to be associated with bone mineral deposition, crystal nucleation, regulation of mineral crystal morphology and in the recruitment, differentiation and maturation of osteoclasts [29,30,31,32,33,34,35,36].

In vivo differences in the bone properties between WT and OC-/- mice were assessed in the mice of different genetic backgrounds and sexes (Table 1). The Karsenty group analyzed female wild-type (WT) and OC-/- mice on a 129/BL6 background, and after 6 months, a significant increase in trabecular bone was observed in OC-/- compared to WT. This increase was also observed in 9 mos. female OC-/- mice [5]. This was due to a large increase in cancellous bone formation as measured by histomorphometry. A significant change in cortical bone formation was observed as well (~60%). No change in bone resorption was observed [5]. These results were corroborated by Dowd et al. in female 6-month-old OC-/- mice (C57BL/6J) [25], where a significant 36% increase in trabecular bone volume/total volume was observed in the knock-out. Using bone biomarkers, they reported a significant 31% increase in serum P1NP, a bone formation marker, in the OC-/- mice and no significant difference in CTX, a bone resorption marker. This is also consistent with micro-CT results on bone from 6 mos. male OC-/- and WT on a C57BL/6J background [10]. They reported an increase in cortical area without an increase in the inner diameter of the OC-/- bone vs. WT, which is attributed to increased bone formation and inhibited resorption [10,37,38]. A study using 6- and 9-month-old females on a C57BL/6N background found no difference in the serum markers of bone formation, P1NP, and bone resorption, CTX, in OC-/- vs. WT mice [3]. However, this study had a limited sample number and large variability in the measured values. The different genetic background could also have affected the result. Hence, except for one study, the majority of the reports show osteocalcin inhibits bone formation without altering bone resorption.

Bone quality is an aspect of bone structure which changes with disease and age. One element of bone quality, material properties, includes compositional properties of the mineral and organic matrix. Techniques which measure bone compositional properties are Fourier transfer infrared imaging and Raman microspectroscopy. These are vibrational spectroscopy imaging techniques which can portray spatial variation in bone tissue compositional properties. Two of the FTIR imaging studies on the OC-/- cortical mineral reported mainly average values across the entire cortical sections containing periosteum, central cortex and endosteum [25,39]. A third study collected Raman spectra only from the central cortex to the endosteum, half the cortical area of the previous two studies, prohibiting the use of these results in the comparison [3]. One study, using the Karsenty mice (129/BL6 background), investigated bone mineral compositional properties using FTIR imaging [39]. One parameter measured, the carbonate to phosphate peak area ratio, is due to amount of total carbonate and phosphate in the hydroxyapatite mineral. Carbonate becomes incorporated into hydroxyapatite whenever it forms in the presence of CO_2_ (body fluids). Total carbonate contains contributions from carbonate substituted in hydroxyapatite for hydroxide (OH, Type A), for PO_4_ (B-type) as well as labile carbonate, which is associated with the apatite surface [39]. Carbonate substitution increases with bone formation and decreases as bone tissue resorbs or dissolves [40]. No significant difference was found in the total carbonate/phosphate ratio between the female 129/B6 OC-/- bone vs. WT. However, this study also analyzed sub-bands of the carbonate and phosphate peak and reported an increase in B-type carbonate in the 6-month-old female OC-/- and a ~35% increased labile carbonate content in the 9-month-old female OC-/- [39]. Dowd et al. reported that female 6 mos. OC-/- (C57BL/6J) cortical bone had a significantly (17%) increased carbonate/phosphate ratio compared to the WT [25]. This study did not analyze the components of the carbonate band, but this result is supportive of the increased carbonate component results reported in the previous study. The increased carbonate is consistent with the increased bone formation rate with no change in resorption reported in the OC-/- mice [5,10,25].

**Figure 4:**
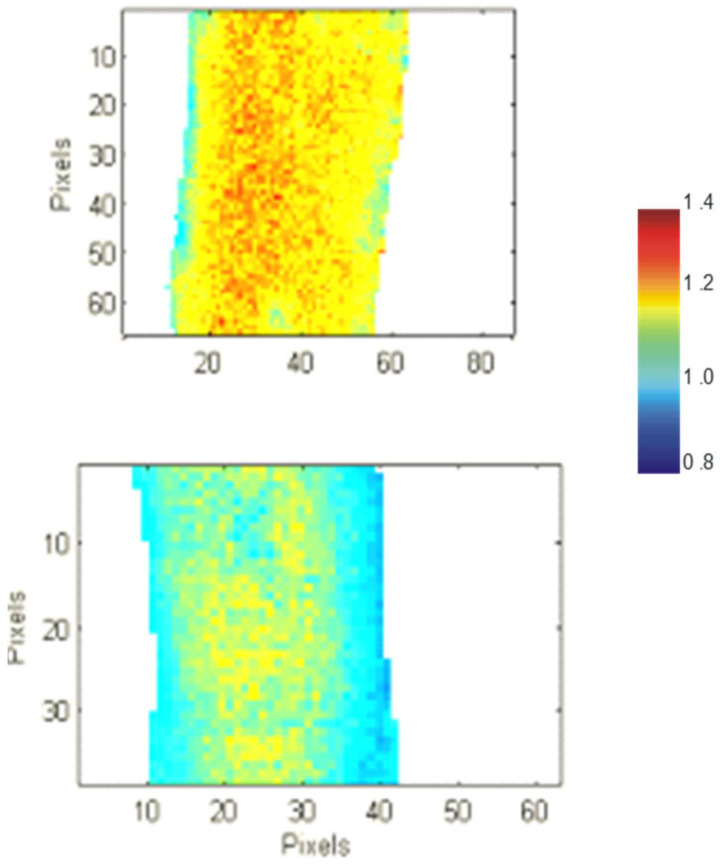
FTIR Image of Cortical Crystallinity across the bone section: Indicative of crystal size and perfection. WT (**top**), OC-/- (**lower**). [25]. Color coded values defined by scale. OC-/- mineral has smaller bone crystals than WT.

Another parameter measured with FTIR imaging was bone crystal size and mineral maturity. The crystallinity parameter was determined from sub-band areas within the phosphate resonance [39] or by the 1030/1020 peak height ratio. This parameter is due to crystal size and the stoichiometric perfection of hydroxyapatite crystal mineral (Ca_10_(PO_4_)_6_(OH)_2_). The 6 mos. female OC-/- mouse on a 129/BL6 background had a significantly reduced bone crystallinity, as did the 6 mos. female OC-/- bones on a C57BL/6J background (~8%) [25,39] (Figure 4). The image in Figure 4 shows a comparison of cortical bone sections from WT and OC-/- displaying color-coded values of the crystallinity parameter. The values corresponding to the colors are shown in the scale. The OC-/- clearly has reduced crystallinity. The increased HPO_4_^2−^ and/or CO_3_^2−^ in the mineral could also contribute to an imperfect crystal and lower this value. In addition, there could be smaller crystal sizes in the OC-/- mineral. Indeed, this was verified by a study using 2D small-angle X-ray scattering, which reported significantly smaller, thinner crystals in the OC-/- mineral in the 6 mos. male C57BL/6J OC-/- mice [9]. The increased bone formation seen in the OC-/- mineral may play a role in the small crystal size reported. Smaller crystals are seen with increased bone formation where crystals are deposited rapidly. This study also reported a significant correlation between crystal thickness and mineral crystal alignment. The mineral crystals were less aligned along the collagen axis in the absence of osteocalcin [9]. This result was later corroborated by a study in female 9 mos. and male 3.5 mos. OC-/- bones from mice on a C57BL/6N background [3].

Mineral maturity can also be assessed with FTIR by comparing acid phosphate (HPO_4_^2−^) substitution in bone mineral between the OC-/- and WT mice. Acid phosphate can substitute into bone hydroxyapatite and is higher in younger, more immature mineral rather than older bone. The imbalance in bone turnover, with increased bone formation over resorption, can lead to an increase in acid phosphate and mineral immaturity as observed in the 6 mos. female OC-/- mineral in the C57BL/6J and 129/BL6 backgrounds [25,39]. 

Although there is agreement in most of the reported bone parameters in the OC-/- on a 129/BL6 and C57BL/6J background, there were some differences reported in cortical mineral. A significant 30% increase in cortical thickness was reported in the 6 and 9 mos. female OC-/- bones on a 129/BL6 background [5]. FTIR analysis of these bones found no difference in the mineral/matrix ratio, which determines the degree of mineralization of the collagen matrix, between the WT and OC-/- 6 mos. female mice [39]. In contrast, no difference in cortical thickness was observed in 6 mos. male or female cortical bones of OC-/- on a C57BL/6J background [10,25]. Also, there was a significant 20% decrease in the mineral to matrix ratio reported in the 6 mos. female C57BL/6J OC-/- bone vs. WT [25]. The difference in results could be due to differences in genetic background between 129/BL6 and C57BL/6J mice. Evidence that bone mass and strength are genetically regulated is provided by association of certain chromosomal loci with high bone mass [41]. The BL6 background was found to have lower bone mineral density and mineral content as compared to some other backgrounds [42] and lower cortical thickness compared to the 129 background specifically [43].

## 4. Bone Biomechanical Measurements

Mechanical testing on the OC-/- mice femora can determine the functional role of osteocalcin in bone strength, stiffness and fracture resistance. Whole-bone strength is affected by geometry, bone mass and tissue material properties [44]. The whole-bone mechanical tests were performed by three-point bending experiments. The femur bone is placed on a stand, and a single loading rate is applied to the whole bone. A monotonically increasing load applied to a bone until it breaks will produce deformations or displacements within the structure [45]. This generates a load vs. displacement curve comprising an initial linear, elastic region before yield where the bone is not permanently deformed. The slope of this linear region provides bone stiffness. With increasing loads, this is followed by a nonlinear plastic region where there is permanent deformation of the bone and determination of the maximum (ultimate load) and the failure point where the whole bone fractures. These are whole-bone structural properties (i.e., femoral diaphysis) [46]. The stiffness parameter describes how much the whole bone deforms when loaded. The maximum load is the greatest load a bone can withstand before fracturing. The failure load is the load causing the bone to fracture, and many times it is the same as the maximum load.

The tissue level (material) properties of bone are independent of bone size and shape and are affected by material microstructure, tissue composition and fracture toughness. The material properties are normalized for known geometric characteristics producing a stress–strain curve where the stress is the load per cross-sectional area and strain is the change in length per initial length [45]. The elastic modulus, (or Young’s modulus) is the tissue-level equivalent to stiffness and also called the tissue-level stiffness. The ultimate stress is the highest load per unit area that the bone tissue can endure before fracturing [46].

Fragility and resistance to fracture (fracture toughness) is another important structural property of bone. In fracture mechanics, this is assessed by measuring the toughness in the presence of a pre-made, substantial crack or flaw [47]. The stress needed to fracture the bone in the presence of this dominant crack is then determined [47]. Femora are notched, wrapped in saline-soaked gauze and then tested in wet conditions with three-point bending. Load–displacement curves are generated and used to measure propagation toughness or resistance to fracture in the presence of this flaw. Fracture toughness is a tissue-level property which is not size-dependent.

Several bone biomechanical measurements were performed on bones from the OC-/- mice. A significantly increased failure load (20%) was reported in 6-month-old female OC-/- on a 129/BL6 background, suggesting a stronger bone [5]. The study did not measure the total or bone area of the cortical bone, nor the Imin, which is the moment of inertia in the plane of bending. The moment of inertia reflects the amount and spatial distribution of bone and is proportional to the periosteal radius and bone size. Normalization with one of these geometric parameters would aid in determining whether the increased failure load (strength) in the OC-/- bones was due to increased bone size or tissue properties vs. WT. A significant 17% increase in maximum load was also found in 6-month-old male OC-/- mice vs. WT on a C57BL/6J background. However, when normalized for size, the difference was eliminated [10]. This indicated the increased cortical and total area enabled OC-/- mineral to compensate for weaker material properties by distributing weight over a larger cross-sectional area. No difference in whole-bone biomechanical properties was reported for the male 6 mos. OC-/- vs. WT on a C57BL/6N background [3]. However, at the micron level using nanoindentation, 3.5 mos. males on this background exhibited a significantly decreased tissue-level stiffness, suggesting weaker tissue properties [3]. A significant 19% decrease in stiffness and a 13% decrease in maximum load was reported for the 6-month-old female OC-/- on a C57BL/6J background. This decrease was not dependent on size since the significant decrease remained when the stiffness and maximum load were size-normalized by their moment of Inertia (Imin) [25]. This indicates the female OC-/- bone is weaker, not due to size but to impaired tissue properties. While the above biomechanical studies report at the macroscale, whole-bone level, a study using nanoindentation reported a 19% significant decrease in tissue-level stiffness (Young’s modulus) in 9 mos. female OC-/- bones (C57BL/6N) at the micron-level scale [3]. At two different size scales, the tissue-level properties of both the female and male OC-/- are weaker than those of the wild type.

Fracture toughness tests were performed on the 6 mos. C57BL/6J males. The results showed that both osteocalcin and osteopontin played a role in energy dissipation by forming dilational bands between mineralized collagen fibers when microdamage was present in bone [26,48]. Upon loading, these bands can rupture, and collagen fibrils separate, forming diffuse damage (submicroscopic cracks) that enables bone tissue to disperse energy without causing fracture [26,48]. The OC-/- mineral had no osteocalcin—thus, no dilational bands could be formed—and less diffuse damage, indicating an increased propensity to fracture. Since fracture toughness tests are independent of bone size, these results further confirm impaired material properties in the OC-/- bone.

In summary, the bone biomechanical results point to impaired or weaker tissue properties in the OC-/- mineral. Tissue properties are size-independent and normalized for size in whole-bone three-point bending data or measured in nanoindentation or fracture toughness experiments. The material or tissue properties are the most significant since they will determine the propensity of the bone to fracture. Weaker tissue properties in the OC-/- mineral were reported for both male and female 6 mos. OC-/- bones (C57BL/6J) [25,26], 3 mos. male and 9 mos. female OC-/- (C57BL/6N) vs. WT containing OC [3]. Weaker tissue properties were reported for both sexes in two different genetic backgrounds. The imbalance in bone turnover could contribute to the weaker tissue properties. The increased bone formation can cause a more immature mineral phase as well as smaller bone crystals, which have been associated with weaker bone mineral [49].

The OC-/- mineral in the 129/BL6 background was reported to have a higher failure load but was never normalized for size nor examined for fracture toughness [5]. Since the tissue-level properties were never determined for OC-/- mineral in this background, we cannot compare with those of other backgrounds. However, it is likely that they may be weaker as well when corrected for size. It was reported the femora on the 129 background had a higher moment of inertia and cortical area vs. B6 femora, and this correlated with higher whole-bone strength (stiffness and maximum load) [43]. The 129 OC-/- femora were reported to have greater cortical thickness than WT [5], so if corrected for size, the difference in strength may disappear. It is possible the OC-/- mineral on a 129 background would have weaker tissue properties if tested for fracture toughness.

## 5. The Role of Osteocalcin in Diabetes and Glucose Metabolism

In addition to its function in bone properties and strength, osteocalcin is reported to function as a hormone affecting glucose metabolism. The undercarboxylated (1 Glu-OC) and uncarboxylated (3 Glu-OC) forms of osteocalcin (Figure 3) were found to be the active form of the hormone [6,7]. Early reports from the Karsenty group [6,7] found 1–6 mos. OC-/- mice on a mixed 129/BL6 background displayed significant glucose intolerance (GTT), insulin resistance (ITT), increased fat pad mass/BW, reduced insulin secretion (GSIS) and β-cell proliferation compared to WT mice. [7]. This was very recently confirmed in 9.5 mos. male OC-/- mice (C57BL/6J) [8]. A significantly increased glucose intolerance, insulin resistance, increased fat pad mass/BW and decreased insulin secretion were reported in male 9.5 mos. OC-/- mice as compared to the WT control [8]. The different onset time, 9.5 mos. vs. 1–6 mos., can be ascribed to the differences in genetic background (C57BL/6J vs. 129/BL6). The small increase in percentage of fat mass/body weight in OC-/- (3.91 +/− 0.38% *g*/*g*) vs. WT (2.6 +/− 0.46% *g*/*g*) with no difference in body weight implies the OC-/- may have a small decrease in lean muscle mass. The OC-/- mice have been reported to have a modest decrease in muscle mass from the age of 6 months on [50]. This small decrease in muscle mass could possibly contribute to glucose intolerance in the 9.5 mos. OC-/- since muscle is involved in glucose uptake.

Further confirmation of the role of OC in glucose metabolism was shown by recovery experiments. It was found that glucose levels were significantly reduced in the OC-/- mice with injection of uncarboxylated OC vs. a vehicle [8]. This was also shown in the initial report from GTT in younger OC-/- mice where injection of glucose and uncarboxylated osteocalcin lowered the blood glucose values as compared to those receiving glucose alone [7]. Additional support comes from a report where intermittent injections of uncarboxylated OC significantly improved glucose tolerance in wild-type mice and mice fed a high-fat diet [51].

In contrast, several years ago, two independent labs could not confirm the role of osteocalcin in glucose homeostasis. One group used the CRISPR/Cas9-mediated gene-editing approach to produce an OC- mouse on a C57BL/6J; C3H background, discussed previously [4]. They found no effect of osteocalcin on glucose metabolism in mice at 6 mos. of age. However, as discussed above, these results cannot be considered since the presence of mutations in the sequence of WT controls was found [27], and it was later discovered that the WT controls used for comparison were obese [28]. The second group investigated the role of osteocalcin in glucose metabolism using 5 and 9 mos. OC-/- mice on a C56BL/6N background. They found no effect of osteocalcin on glucose metabolism nor insulin resistance at either age, 5 or 9 months [3]. This is in disagreement with the previous study on 9.5 mos. males (C57BL/6J), which found a significant effect on glucose intolerance and insulin resistance in the OC-/- vs. WT as tested by the GTT and ITT [8]. The blood glucose values in the GTT for the 9-month-old C57BL/6N OC-/- mice were non-significantly higher than WT [3], and this was probably due to the lower glucose administered to the mice (1 g/kg BW) as compared to the other two studies, which used 2 g/kg/BW [7,8]. Examining the data in the ITT of the C57BL/6N OC-/- mice [3] shows that the insulin value given was too low since it did not even lower the blood glucose of the WT mice. The dose of insulin is chosen by the amount which will lower blood glucose in the WT to ~50% of its initial value. That dosage is then applied to the OC-/- mice.

Too low an insulin dose would obscure an effect in the OC-/- if it were present. Thus, improper dosing of both glucose (GTT) and insulin (ITT) may have been responsible for the disagreement in the results [3].

The role of osteocalcin in glucose homeostasis has been confirmed in several careful studies [6,7,8]. The glucose intolerance and reduced glucose stimulated insulin secretion in the OC-/- mice, which was supported by smaller pancreatic islets, reduced insulin expression in β cells and a decrease in β cell proliferation in the absence of osteocalcin [7,52]. Insulin secretion was shown to be regulated by the interaction of the active forms of the hormone, uncarboxylated (3 Glu-Oc) (Figure 3) or undercarboxylated (1 Glu-OC) [6,7], with the *Gprc6a* receptor [53] in the pancreas.

**Figure 5:**
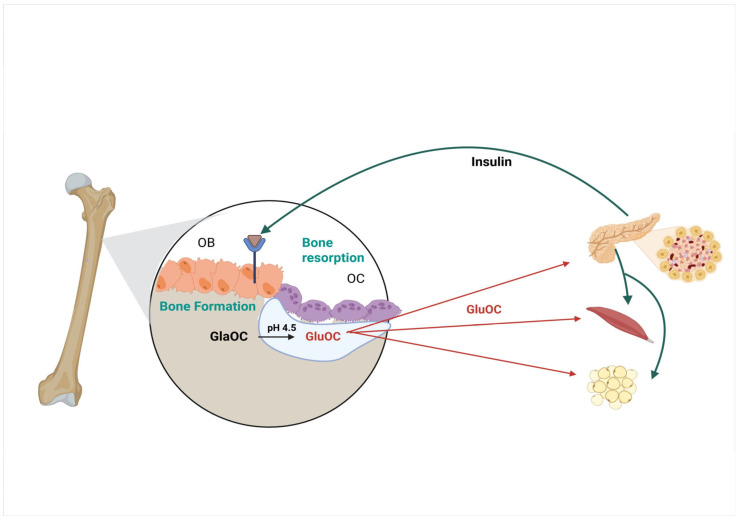
The proposed model for insulin signaling. The proposed model for insulin signaling which produces decarboxylation of osteocalcin [6]. Insulin binding to the receptor on the osteoblast (OB) causes a decrease in osteoprotegerin expression leading to an increase in bone resorption by osteoclasts (OC). The acidification of the bone extracellular matrix promotes osteocalcin decarboxylation. The production of the active form of osteocalcin (GluOC) results in β-cell proliferation and insulin secretion in the pancreas and increased insulin sensitivity.

Insulin signaling in osteoblasts was proposed to explain the integration of bone remodeling and energy metabolism [6] (Figure 5). Insulin binds to the insulin receptor on osteoblasts which destabilizes FOXO1 and reduces its ability to activate Osteoprotegerin, an inhibitor of osteoclast function. Bone resorption is then stimulated, and 3-Gla OC is released from the bone matrix and decarboxylated, releasing the active form of the hormone (1-Glu OC) [6]. It has been shown that acidic pH can decarboxylate proteins [54] and later shown that pH 4.5 and the resorptive activity of osteoclasts could activate (decarboxylate) osteocalcin [6]. The 1-Glu-OC then interacts with the GPRC6A receptor on the pancreas, inducing insulin production and release by β cells.

Previous studies have shown no significant reduction in bone resorption in the OC-/- vs. WT mouse bone. However, total inactivation of the insulin receptor in the osteoblast produced a small reduction in serum CTX, a marker of bone resorption [6]. If total inactivation of the insulin receptor produced a small reduction, then it is unlikely a mild reduction in the circulating hormone could produce a detectable decrease in bone resorption. An effect on bone resorption might be observed if measurements are collected immediately after feeding when insulin is released vs. at random.

Osteocalcin has also been shown to interact with the GPRC6A receptor on the intestinal endocrine cells, increasing pancreatic β cell function and insulin secretion by activating the release of GLP-1 [55,56,57]. Secreted insulin can then react with insulin-sensitive tissues as well as with the receptor on the osteoblast to produce more 1 Glu-OC [6]. Additionally, the active form of osteocalcin has also been demonstrated to interact with the GPRC6A receptor in muscle [50] and in adipocytes [6,7], which are covered in other reviews.

We have shown that when comparing recently published results, justifiable comparisons and appropriate experimental studies, there is a significant amount of consensus concerning the role of osteocalcin in bone properties and glucose metabolism in the OC-/- mouse studies. Results collected from the same anatomical area show similar bone mineral properties; biomechanical measurements reporting tissue-level properties show weaker material properties in the OC-/-, and a recent OC-/- mouse study with proper dosing confirmed the role of osteocalcin in glucose metabolism.

Several studies have indicated an involvement of osteocalcin in glucose metabolism and diabetic complications in humans. Human pancreatic islets which were implanted in mice increased insulin production after exogenous osteocalcin injection [58]. Increased glucose, insulin and triglyceride levels were found in population studies where a polymorphism in the GPRC6a gene was present [59]. Human as well as rodent studies have shown a role of undercarboxylated osteocalcin in glucose metabolism. Decreased undercarboxylated osteocalcin was reported in diabetics and prediabetics [60], and serum osteocalcin levels are inversely related to fasting glucose, insulin and BMI in older men [61,62]. Elevated bone fracture risk is reported in type 2 diabetes and cannot be explained by bone mineral density [63,64,65]. Reduced osteocalcin levels could very well contribute to the higher propensity for bone fracture in this disease by contributing to weaker bone tissue properties and reduced dilational band formation [26].

More studies on the role of osteocalcin in glucose regulation are needed in humans as the data is limited and much of it associative in nature. However, showing cohesive evidence in mouse studies is a good start and encourages more research in this area. In this review, we see that osteocalcin plays a role in bone mineral properties, preventing bone fracture and in glucose regulation. It is a molecule affecting both symptoms of diabetes and some symptoms seen in aging, and hopefully one day may be of therapeutic use.

## Figures and Tables

**Table 1:** Comparison of bone mineral properties from OC-/- vs. WT mice from different groups. The arrows signify that the parameter is significantly increased (↑), decreased (↓) or not significantly different (↔) in the OC-/- mice as compared to the WT. * The N/A (not applicable) is due to obesity reported in the WT mice [28], so no comparison can be made with the WT vs. OC-/- from this study (see text). ND—not determined. Not comparable refers to data not collected in the same region of the bone.

Parameter	Karsenty	Karsenty/ Boskey	Dowd	Vashishth	Komori	Williams
Genetic background	129/BL6	129/BL6	C57BL/6J	C57BL/6J	C57BL/6N	C57BL/6J/C3H
Age	6 mos.	6 mos. 9 mos.	6 mos.	6 mos.	6 or 9 mos.	N/A *
Sex	Female	Female	Female	Male	M and F	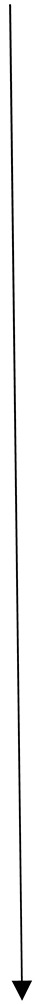
Cortical thickness	**↑**		**↔**	**↔**		
Trabecular bone	**↑**		**↑**	**ND**	**↔**	
Bone formation	**↑**		**↑**	**↑**	**↔**	
Bone resorption	**↔**		**↔**	**ND**	**↔**	
Carbonate/P		**↔**	**↑**	**ND**	**Not** **comparable**	
Labile CO_3_^2−^		**↑**		**ND**		
B-CO_3_^2−^		**↑**		**ND**		
Acid phosphate		**↑**	**↑**	**ND**	**Not** **comparable**	
Mineral/matrix		**↔**	**↓**	**ND**	**Not** **comparable**	
Crystal size		**↓**	**↓**	**↓**	**Not comparable**	
Crystal thickness				**↓**		
Crystal alignment				**↓**	**↓**	

## Data Availability

No new data were created or analyzed in this study. Data sharing is not applicable to this article.
